# Impact of Clinical Practice Restriction on Medical Students’ Perceptions of Simulation Training: A Retrospective Cohort Study

**DOI:** 10.1007/s40670-025-02568-5

**Published:** 2025-11-17

**Authors:** Koichiro Abe, Takatsugu Yamamoto, Hirotoshi Kikuchi, Ichiro Kaneko, Kumiko Konno, Shoko Horita, Yumiko Okubo

**Affiliations:** 1 Department of Medicine, Teikyo University School of Medicine, Tokyo, Japan; 2 Department of General Medical Education and Research Center, Teikyo University, Tokyo, Japan; 3 Teikyo Simulation Education and Research Center, Teikyo University, Tokyo, Japan; 4 Department of Medical Education, Teikyo University School of Medicine, Tokyo, Japan

**Keywords:** Simulation training, Medical education, Medical student, Student’s perception

## Abstract

The coronavirus disease 2019 pandemic forced medical education to adopt alternative programs focused on infection prevention. Simultaneously, simulation training gained importance as a tool to complement clinical experience. This study aimed to compare medical students’ perceptions regarding the effectiveness of simulation training between those who participated during COVID-19-related clinical practice restrictions and those in the post-restriction period. Fifth-year medical students from academic year (AY) 2022 (during restrictions) and AY 2023 (after restrictions) completed a survey before and after simulation training in general internal medicine. The survey assessed students’ perceptions of effectiveness in knowledge, skills, motivation, communication, team healthcare, and clinical experience using a 5-point Likert scale. Simulation training significantly enhanced students’ perceptions across all survey domains regardless of clinical practice restrictions. The most notable improvement was observed in team healthcare awareness, which increased from 3.9 to 4.8 in both cohorts. In contrast, perceived contribution to clinical experience remained lower than other domains, with only modest increase in both AY 2022 (3.2 → 3.4) and AY 2023 (3.4 → 3.7). This study found that medical students perceived simulation training as effective for enhancing their learning in clinical competencies, but felt its role in replicating real patient experience was limited.

**Supplementary Information:**

The online version contains supplementary material available at 10.1007/s40670-025-02568-5.

## Introduction

Clinical practice in medical schools has at times been restricted due to external circumstances. The coronavirus disease 2019 (COVID-19) pandemic had a significant impact on clinical medical education worldwide [1, 2]. During this period, many medical schools around the world were compelled to modify their curricula and implement alternative programs such as simulation-based education and online learning to reduce infection risks [3–6]. Although face-to-face and team-based learning have been associated with higher learner satisfaction and improved outcomes [7], medical students faced severe limitation on direct patient contact, leading to a loss of authentic clinical experience. Under such conditions, simulation-based training emerged as a key educational tool to complement real clinical practice [8–10].

Simulation training offers several advantages. For learners, the simulation environment provides less psychological pressure, allowing them to try new or challenging procedures [11]. They can practice repeatedly and mistakes, which would be unacceptable in actual clinical settings, can be tolerated as learning opportunities in the simulation environment. Additionally, they can receive effective feedback from instructors quickly. Simulation-based medical education conducted with high fidelity under appropriate environments have been proven to improve clinical performance, including their skills and behavior [12–16]. However, the educational impact of simulation may vary depending on students’ motivation and the surrounding learning environment, both of which can be influenced by contextual factors such as limited patient exposure [17].

Although the benefits of simulation training are well documented, few studies have directly compared students’ perceptions of its effectiveness under different levels of clinical exposure. We assumed that medical students, who had restricted clinical practice opportunities, would have higher expectations for simulation training. This study therefore aimed to compare medical students’ perceptions of simulation training effectiveness between two adjacent cohorts: one that undertook simulation during a period of restricted clinical practice, and another after restrictions were lifted.

## Materials and Methods

### Study Design and Participants

This study was a single-center retrospective cohort study that summarizes the results of a questionnaire survey on simulation training conducted with fifth-year medical students at Teikyo University School of Medicine during their rotation in general internal medicine department in the academic year (AY) 2022 and AY 2023. Clinical clerkships for fifth-year students at our university are conducted from March to the following February. Since March 2020, our university has transitioned to alternative programs as part of the COVID-19 infection control measures. The duration of clinical practice has been shortened, lectures have mostly transitioned to online platforms like Zoom and Teams, and direct patient encounters have been restricted. With the decrease in the number of infections and the establishment of infection control measures, we resumed clinical practice without any restrictions starting from the AY 2023. In both AY 2022 and AY 2023, students participated in the same simulation training program in general internal medicine. For AY 2023 students, this training was conducted in combination with full in-person clinical practice. The timing of the simulation training depended on when each student rotated through the general internal medicine department. Therefore, the amount of clinical experience prior to the simulation varied among students. We collected questionnaires from medical students who participated in the simulation training. There were no exclusion criteria for this study.

### The Procedures of Simulation Practice

A Resusci Anne simulator (Laerdal Medical, Stavanger, Norway) equipped with functions for auscultating respiratory sounds and palpating pulses was used in the simulation practice. We prepared three scenarios: anaphylactic shock, septic shock, and acute coronary syndrome. The medical students were presented with simulated patients exhibiting dyspnea, fever, and chest pain, without disclosure of the underlying diagnosis, and were instructed to make a diagnosis based on the medical history, physical examination findings, and diagnostic test results. Each scenario was designed with specific clinical variations to simulate realistic situations. In the anaphylaxis scenario, the facilitator randomly selected one of several possible triggers such as a bee sting, buckwheat, nonsteroidal anti-inflammatory drugs, or contrast media, and presented a corresponding case setting. Students were assessed on their ability to identify the causative agent through history taking, make a correct diagnosis, distinguish characteristic auscultatory findings, as the simulator’s breath sounds were set to stridor, and administer intramuscular adrenaline at a dose of 0.3 mg. In the septic shock scenario, the facilitator randomly selected one of three underlying infections: pyelonephritis, acute cholecystitis, or pneumonia. All cases involved patients with diabetes mellitus and poor adherence to oral medications. Students were evaluated on their ability to elicit key physical findings, including costovertebral angle tenderness in pyelonephritis, Murphy’s sign in acute cholecystitis, and coarse crackles in the right lower lung field in pneumonia. They were then asked to identify appropriate diagnostic tests. The facilitator provided corresponding test results, including Gram staining of urine culture, showing gram-negative rods, for pyelonephritis, abdominal ultrasonography for acute cholecystitis, and chest radiography with Gram staining of sputum, showing gram-positive diplococci, for pneumonia. Students were assessed on their interpretation of these findings and the appropriateness of their diagnoses. In the acute coronary syndrome scenario, the simulated patient had a history of diabetes, hypertension, and smoking. Students were evaluated on their ability to conduct appropriate history taking to diagnose acute coronary syndrome and to identify the culprit coronary artery based on electrocardiographic findings (ST elevation). Three different electrocardiograms corresponding to each culprit artery (right coronary, left anterior descending, and left circumflex) were prepared for this scenario.　The scenario then progressed to ventricular fibrillation or pulseless ventricular tachycardia, during which students were required to diagnose the rhythm on the monitor and were assessed on their ability to perform cardiopulmonary resuscitation in accordance with the established algorithm. Across all scenarios, students were required to initiate essential emergency management procedures for critically ill patients, including attaching electrocardiogram monitor and pulse oximeter, oxygen supplementation, and securing intravenous access. Each group consisted of around ten students. Initially, we formed three small groups of three or four students. Within each small group, students determined who would serve as the leader for each of the three simulation scenarios. Because there were three scenarios, groups of four students assigned two leaders to the septic shock. Depending on the group size, one or two leaders took the medical history from the facilitator playing the role of the patient and performed physical examinations on the simulator. Then, the group members considered the necessary tests and, based on the test results presented by the facilitator, made diagnoses and provided treatment. While one small group conducted the simulation, the other groups observed the session. After the session, the facilitator conducted debriefings on knowledge, skills, and attitudes based on the Gather–Analyze–Summarize model. A total of nine sessions were conducted, as each of the three small groups performed all three scenarios. The duration of each session, including debriefing, was set at approximately 15 min. The facilitator modified the background of the scenario to avoid monotony. All simulation sessions were facilitated by the same instructor, who held board certification in internal medicine from the Japanese Society of Internal Medicine and was qualified as a director of the Japanese Medical Emergency Care Course. The content of the simulation scenarios and the evaluation criteria were consistent between AY 2022 and AY 2023.

### Questionnaire

The components of questionnaire assessed students’ self-perception of knowledge improvement (knowledge), clinical skills (skill), motivation increase (motivation), communication skill (communication), recognition of the importance of team-based healthcare (Team healthcare), and complement of clinical experience (clinical experience). To investigate changes in perceptions of simulation training, the same survey was conducted before and after the training (Table 1). Medical students completed the pre-survey just before the start of the simulation session and the post-survey immediately after its completion. Each component was evaluated using a single-item question on a 5-point Likert scale, with 5 being ‘strongly agree’ and 1 being ‘strongly disagree’. The same items were used in both AY 2022 and AY 2023 cohorts.

**Table 1:** Survey items assessing students’ perceptions before and after simulation training

Before training
1. I think the simulation training will help improve my clinical knowledge
2. I think the simulation training will help improve my clinical skills
3. I think the simulation training will help increase my motivation to learn
4. I think the simulation training will help improve my communication skills
5. I think the simulation training will help me recognize the importance of team-based healthcare
6. I think the simulation training will provide an experience comparable to actual clinical practice
After training
1. Through the training, I feel that my clinical knowledge has improved
2. Through the training, I feel that my clinical skills have improved
3. Through the training, I feel that my motivation to learn has increased
4. Through the training, I feel that my communication skills have improved
5. Through the training, I recognized the importance of team-based healthcare
6. Through the training, I felt that the experience was comparable to actual clinical practice

### Statistics

All statistical analyses were performed using SPSS Statistics version 28 (IBM Japan, Tokyo, Japan). The questionnaire items used a 5-point Likert scale, and the data were summarized as mean ± standard deviation, which is common in educational research with large samples. Given the large sample size in this study, treating Likert scores as continuous variables was considered acceptable based on the central limit theorem. More detailed data (medians, ranges, and modes) were provided in supplement 1. A paired t-test was used to compare the medical students’ awareness before and after the simulation training for both AY 2022 and AY 2023. The effect size was measured by Cohen’s d and presented as an absolute value. The comparison of medical students’ perceptions after simulation training between AY 2022 and AY 2023 was calculated using a two-tailed t-test. Differences with two-sided alpha levels of < 0.05 were determined as statistically significant.

### Ethics

This study was approved by the ethics committee of Teikyo University (approval number 23–043-2). All procedures followed relevant guidelines and regulations. The need to obtain informed consent was waived by the ethics committee that approved the study, given the retrospective design of the study.

## Results

In AY 2022, 121 fifth-year medical students participated in the simulation training, and 119 of them (68 males and 51 females) completed the survey. In AY 2023, all 113 participants (68 males and 45 females) completed the survey. In AY 2022, there were two non-respondents, but the reasons were not reported. Table 2 shows the changes in perceptions of simulation training before and after the sessions.

**Table 2:** Comparison of students’ perceptions before and after simulation training in AY 2022 (during restrictions) and AY 2023 (after restrictions)

	AY 2022						AY 2023					
Component	Mean ± SD		Change (After -Before)	95% CI	*P*-value*	Cohen’s d	Mean ± SD		Change (After -Before)	95% CI	*P*-value*	Cohen’s d
	Before	After					Before	After				
Knowledge	4.2 ± 0.56	4.7 ± 0.47	0.5	0.41–0.65	< 0.001	0.83	4.3 ± 0.60	4.7 ± 0.49	0.4	0.26–0.49	< 0.001	0.60
Clinical skills	4.2 ± 0.60	4.6 ± 0.55	0.4	0.27–0.50	< 0.001	0.61	4.4 ± 0.56	4.7 ± 0.48	0.3	0.20–0.41	< 0.001	0.53
Motivation	3.9 ± 0.73	4.5 ± 0.58	0.6	0.45–0.72	< 0.001	0.78	4.1 ± 0.77	4.6 ± 0.57	0.5	0.31–0.59	< 0.001	0.61
Communication	3.9 ± 0.84	4.3 ± 0.80	0.4	0.30–0.60	< 0.001	0.55	3.9 ± 0.84	4.4 ± 0.75	0.5	0.29–0.65	< 0.001	0.50
Team healthcare	3.9 ± 0.78	4.8 ± 0.39	0.9	0.84–1.11	< 0.001	1.34	3.9 ± 0.78	4.8 ± 0.38	0.9	0.76–1.05	< 0.001	1.14
Clinical experience	3.2 ± 0.96	3.4 ± 0.97	0.2	0.001–0.32	0.048	0.18	3.4 ± 0.90	3.7 ± 0.97	0.3	0.04–0.38	0.018	0.23

*AY* Academic year, *SD* Standard deviation, *CI* Confidence interval

*A paired t-test was used

**Fig. 1:**
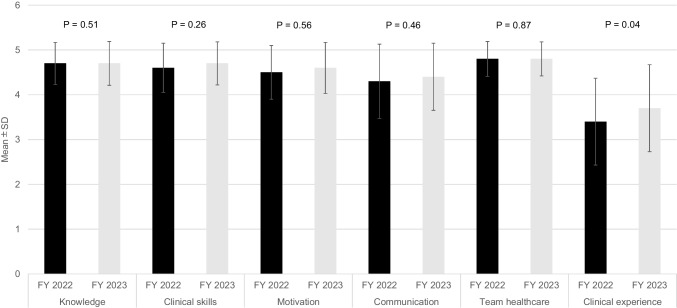
Comparison of medical students’ perceptions after simulation training between AY 2022 and AY 2023. Each bar represents the mean post-training score on a 5-point Likert scale across six domains. Error bars indicate standard deviation. AY: academic Year, SD: Standard deviation

In both AY 2022 and AY 2023, medical students perceived significant improvement in knowledge, clinical skills, motivation, communication, team healthcare, and clinical experience after simulation training. The effect sizes for clinical experience were smaller than those for the other domains in both cohorts. Particularly in team healthcare, students’ perceptions showed the most significant increase before and after the simulation training in both AY 2022 and AY 2023. On the other hand, students felt that the contribution to clinical experience was lower compared to other components, both in AY 2022 and AY 2023. Figure 1 shows a comparison of perceptions after simulation training between AY 2022 and AY 2023. There were no significant differences in knowledge, clinical skills, motivation, communication, and team healthcare. However, regarding the clinical experience, medical students in AY 2022, who had restricted clinical practice, perceived it less favorably than those in AY 2023.

## Discussion

This study explored changes in medical students’ self-perceived effectiveness of simulation training and found improvements across multiple domains, regardless of clinical practice restrictions. Simulation-based education has been shown in numerous reports to enhance medical students’ knowledge, skills, and motivation, and to contribute to increased confidence in their own abilities [18–22]. In a randomized controlled study comparing satisfaction with simulation training and group discussion, medical students found simulation training to be more enjoyable, stimulating, and satisfying [23].

In our study, the most significant improvement in students’ awareness before and after the simulation training was in team healthcare (Table 2). Other studies have also shown that simulation training is effective in enhancing communication skills and teamwork [19, 24]. Communication skills and teamwork are among the most important components in clinical settings [25–27]. Improving these can not only enhance team performance but also lead to a reduction in errors [28]. These results suggest that simulation training can be expected to improve the overall clinical competencies of medical students.

Regarding clinical experience, although there was improvement in perception before and after simulation training in both AY 2022 and AY 2023, the absolute value was lower compared to other domains (Table 2). The effect sizes for clinical experience were also small, which suggests that the perceived effects were limited. Moreover, medical students in AY 2022 perceived the enhancement of their real clinical experience through simulation training to be lower compared to the students in AY 2023. Due to the lack of face-to-face training sessions and limited real clinical experience in AY 2022, it may have been challenging for medical students to accurately evaluate their experiences. According to Ten Eyck et al., medical students perceived that simulation training provided a more realistic clinical experience compared to group discussions [23]. However, this result was comparative and may not reflect an absolute evaluation of the simulation training itself. In simulation training, simplified scenarios are prepared to achieve specific learning outcomes. However, in real clinical practice, even the same disease can present with atypical medical history, physical findings, and test results, making a definitive diagnosis difficult. Therefore, there are aspects where simulation training cannot fully complement the experience of actual clinical practice. Kneebone et al. argued that excessive simplification in simulation learning can make it more difficult to adapt to the complexities encountered with real patients [29]. Scott et al. created scenarios with confused medical histories, lacking clear diagnoses and diagnostic tests, to replicate the uncertainties of real clinical practice. While medical students tended to feel disappointed by the lack of definitive diagnoses and expected clear answers, they found the simulation beneficial as it provided an opportunity to develop strategies for dealing with uncertainties [30]. Involvement of excessive elements of uncertainty in simulation training might lead to confusion among medical students. However, with adequate learning of typical cases and appropriate feedback, the inclusion of uncertainty can potentially make simulation training closer to real clinical practice.

In the comparison of post-simulation perceptions between AY 2022 and AY 2023, students in AY 2023 scored higher in the domain of clinical experience. However, as mentioned previously, AY 2022 students had already shown lower baseline scores than those in AY 2023. Therefore, the interpretation of post-simulation differences between academic years should be made with caution, as baseline differences may have influenced the outcomes. A speculative explanation for the lower expectations among AY 2022 students is that various restrictions during clinical practice may have caused stress for medical students, leading to decreased motivation and concentration.

This study was limited by its retrospective design and single-institution setting. In addition, comparisons were made between students from only one academic year per group. As such, the findings should be interpreted with caution, given the potential influence of cohort-specific characteristics alongside the impact of clinical practice restrictions. Furthermore, this study relied on students’ self-perceived evaluations, which are inherently subjective and may not correspond precisely to their actual level of knowledge or skills. Moreover, each of the six domains in the questionnaire was assessed using a single-item measure, which may limit the reliability of the evaluation. Finally, due to the pre-/post-test design, it was not possible to fully control for variations in students’ prior learning experiences before participating in the simulation training.

## Conclusions

Medical students perceived simulation training to be an effective educational tool, regardless of whether clinical practice was restricted. The impact of simulation training on team healthcare was particularly notable. However, efforts are needed to bridge the gap between simulation and authentic clinical experience. To achieve this, it is important to implement simulation training across multiple clinical departments and incorporate a variety of clinical scenarios. Exposure to simulations in diverse settings may increase the likelihood that medical students will encounter similar situations in actual clinical practice. Such experiences may help them recognize the continuity between simulated and real clinical environments.

## Supplementary Information

Below is the link to the electronic supplementary material.

Supplementary file1 (PDF 114 KB)

## Data Availability

The dataset is available at Zenodo: 10.5281/zenodo.15710582.
