# Supplementary material for: Impact of Clinical Practice Restriction on Medical Students’ Perceptions of Simulation Training: A Retrospective Cohort Study
Source: Med Sci Educ. 2025 Nov 17;36(1):287–93. doi: 10.1007/s40670-025-02568-5 (PMC13043958; doi:10.1007/s40670-025-02568-5)
Supplement: Supplementary file 1 — Supplementary file1 (PDF 114 KB) [file 40670_2025_2568_MOESM1_ESM.pdf]

Supplement Table 1. Descriptive statistics of students' perceptions (mean, median, range, and mode) before and after simulation training in AY 2022 (during restrictions) and AY 2023 (after restrictions)

AY 2022								
Component	Mean		Median		Range (Min-Max)		Mode	
	Before	After	Before	After	Before	After	Before	After
Knowledge	4.2	4.7	4	5	2-5	3-5	4	5
Clinical skills	4.2	4.6	4	5	2-5	3-5	4	5
Motivation	3.9	4.5	4	5	2-5	3-5	4	5
Communication	3.9	4.3	4	4	2-5	1-5	4	5
Team healthcare	3.9	4.8	4	5	2-5	3-5	4	5
Clinical experience	3.2	3.4	3	3	1-5	1-5	3	3

AY 2023								
Component	Mean		Median		Range (Min-Max)		Mode	
	Before	After	Before	After	Before	After	Before	After
Knowledge	4.3	4.7	4	5	2-5	3-5	4	5
Clinical skills	4.4	4.7	4	5	3-5	3-5	4	5
Motivation	4.1	4.6	4	5	1-5	3-5	4	5
Communication	3.9	4.4	4	5	2-5	2-5	4	5
Team healthcare	3.9	4.8	4	5	2-5	4-5	4	5
Clinical experience	3.4	3.7	4	4	2-5	1-5	4	4

AY: Academic year
